# Advances of Long Non-Coding RNA in Perennial Plants: Development and Stress Responses

**DOI:** 10.3390/plants14213280

**Published:** 2025-10-27

**Authors:** Yayu Guo, Danni Guo, Yunqing Wang, Jinhuan Yin, Guijun Liu, Huimin Xu

**Affiliations:** 1 College of Biological Sciences, China Agricultural University, Beijing 100193, China; gdn@cau.edu.cn (D.G.); 2022302050102@cau.edu.cn (Y.W.); 2 Oak Research Center, College of Forestry, Beijing Forestry University, Beijing 100083, China; guoyau1510@bjfu.edu.cn; 3 Natural History Museum of China, Beijing 100050, China; 4 Beijing Academy of Science and Technology, Beijing 100089, China; 5 State Key Laboratory of Tree Genetics and Breeding, College of Biological Sciences and Technology, Beijing Forestry University, Beijing 100083, China; yinjinhuan@bjfu.edu.cn

**Keywords:** long non-coding RNA, perennial plants, abiotic and biotic stress, regulatory mechanisms

## Abstract

Long non-coding RNAs (lncRNAs), which have RNA transcripts over 200 nucleotides long and do not usually code for proteins, play an important role in plant growth and development, by interacting with multiple signaling pathways and participating in the regulation of fundamental biological processes. This review systematically summarizes the classification and regulatory mechanisms of plant lncRNAs, with a focus on recent advances in research on their regulatory roles in woody plant growth, development, and responses to abiotic and biotic stresses. Additionally, we discuss the current challenges in lncRNA research on plant ontogeny and stress responses, aiming to provide a theoretical foundation for future studies on lncRNAs in perennial plants.

## 1. Introduction

Long non-coding RNAs (lncRNAs) are defined as RNA transcripts longer than 200 nucleotides (nt) that usually have no protein-coding potential or lack an open reading frame (ORF) encoding >100 amino acids [1]. They typically have a low exon number, retain intronic sequences, and lack functional ORFs, an initiation codon, and a termination codon. As a result, lncRNA transcripts are generally not translated into proteins, representing a fundamental difference between lncRNA and messenger RNA (mRNA) transcripts. However, lncRNAs function as key transcriptional regulators, playing crucial roles in various plant biological processes, including growth and developmental programs, stress response mechanisms, and epigenetic regulation. Their direct involvement in epigenetic regulation underpins key functions in plant morphogenesis, developmental transitions, and environmental adaptation [2,3,4,5]. The increasing availability of literature on long non-coding RNAs (lncRNAs) in perennial plants has prompted this review, which focuses on advances in woody species.

**Figure 1:**
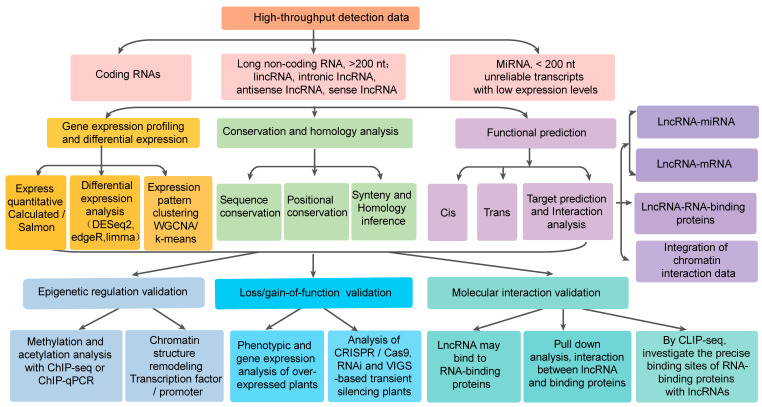
Functional research and analysis methods of long non-coding RNA.

Plant lncRNAs are primarily transcribed by RNA polymerase II from the sense and antisense genomic strands. As highlighted by St. Laurent et al. [6], these molecules exhibit remarkable structural and functional heterogeneity across classification systems. In plants, functionally categorized by genomic context, they comprise long intergenic ncRNAs (lincRNAs) derived from intergenic regions, intronic ncRNAs (incRNAs) originating within introns, and natural antisense transcripts (*NATs*) transcribed from the DNA strand located opposite sense or protein-coding genes—collectively representing conventional linear lncRNAs. In plants, a significant portion of lncRNAs, particularly long intergenic non- lincRNAs, originate from transposable elements (TEs) or contain TE sequences. The rapid evolution of TE-lncRNAs facilitates plants’ adaptation to variable environments [6]. Additionally, circular RNAs (circRNAs) constitute a structurally distinct class, typically forming covalently closed loop structures through back-splicing of exons or introns (or both). Each lncRNA subtype follows unique biogenesis pathways [7], giving them distinct regulatory capabilities that operate through *cis*- or *trans*-acting mechanisms (Figure 1). Further classification distinguishes polyadenylated [poly(A)+] and non-polyadenylated [poly(A)−] isoforms based on post-transcriptional processing [8].

## 2. Regulatory Mechanisms of Plant lncRNAs

**Figure 2:**
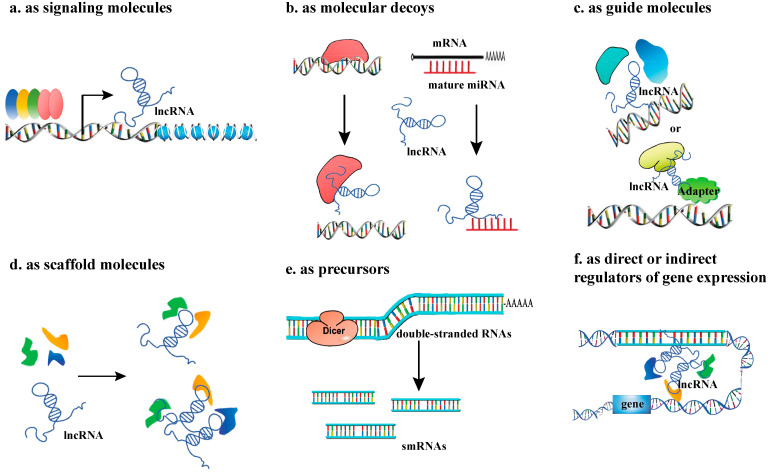
The regulatory mechanisms of IncRNAs. (**a**) lncRNA as signaling molecules; (**b**) lncRNA as molecular decoys; (**c**) lncRNAs as guide molecules; (**d**) LncRNAs as scaffold molecules, making proteins into a complex or spatial proximity; (**e**) lncRNAs as precursors; (**f**) lncRNAs as direct or indirect regulators of gene expression.

Although lncRNAs do not code for proteins, they exhibit regulatory functions at epigenetic, transcriptional, and post-transcriptional levels through diverse molecular mechanisms. There is increasing evidence that lncRNAs are essential regulators of a wide range of biological processes and function through diverse mechanisms (Figure 2).

### 2.1. LncRNAs as Signaling Molecules

The classification of lncRNAs as ‘signaling molecules’ encompasses those with critical functions in signaling pathways. This is a functional classification, and the underlying molecular mechanisms can be diverse. As signaling molecules, lncRNAs can respond to different stimuli, sense the cellular environment, and participate in the transduction of special signaling pathways, thus regulating the expression of target genes. Some lncRNAs have conserved motifs that can be recognized by other regulators. These lncRNAs are differentially expressed under various emergency stimuli, thus integrating stress signaling pathways. In *Arabidopsis thaliana*, stress-associated expression patterns of the lncRNAs were systematically assayed, which were supported by specific binding of stress-related transcription factors (TFs), such as phytochrome-interacting factor 4 (PIF4) and PIF5 [8].

### 2.2. LncRNAs as Molecular Decoys

As bait molecules, lncRNAs can act as competitive endogenous RNAs (ceRNAs) to regulate the expression of target genes by recruiting other RNA-binding proteins. In addition, LncRNAs participate in forming regulatory networks by competitively binding miRNAs, a function analogous to that of circRNAs [9,10]. In addition, lncRNAs can act as endogenous miRNA target mimics (eTMs) to regulate the function of miRNAs by binding to miRNAs through complementary sequences, thereby inhibiting miRNA interactions. For example, the rice lncRNA MIKKI directly traps miRNA171 through complementary base pairing, blocking mRNA binding between miRNA171 and its target gene SCL (whose deficiency inhibits taproot elongation), which increases the expression level of the SCL gene, thereby promoting taproot growth [11].

### 2.3. LncRNAs as Guide Molecules

Acting as guides for RNA-binding proteins, lncRNAs bind or localize to regulatory sites to perform gene regulation [12], usually through specific spatial structures rather than specific sequences [13]. In rice (*Oryza sativa*), antisense lncRNA *LAIR*, which is transcribed from the antisense strand of the leucine-rich repeat receptor kinase (*LRK*) gene cluster, directly binds the histone modified proteins OsMOF and OsWDR5, causes them to become enriched in the *LRK1* gene region, promotes the H3K4me3 and H4K16ac modifications in this gene region, and upregulates the expression of the *LRK1* gene [14].

### 2.4. LncRNAs as Scaffold Molecules

Usually, a lncRNA molecule contains multiple domains that can bind to different proteins or other effector molecules, so it can serve as a central platform for complex assembly [15]. In *Arabidopsis thaliana*, APOLO is a lncRNA located at 5148 bp upstream of the *PID* gene, and is a guide and scaffold molecule involved in the regulation of lateral root development as a guide and scaffold molecule. APOLO acts as a scaffold RNA in the formation of chromatin loops, forming an R-loop structure by complementing its neighboring *PID* gene region with short sequences to regulate the dynamics of chromatin loops [16].

### 2.5. LncRNAs as Precursors

LncRNAs can act as precursors for various small RNAs, including small interfering RNAs (siRNAs), microRNAs (miRNAs), and PIWI-interacting RNAs (piRNAs). Most miRNAs are processed from long primary transcripts (pri-miRNAs), and certain lncRNAs have been identified as precursors for siRNAs or miRNAs [17]. Similarly, piRNAs, which are crucial for transposon silencing, are also derived from lncRNA precursors [18].

### 2.6. LncRNAs as Direct or Indirect Regulators of Gene Expression

LncRNAs can directly affect gene transcription and translation by complementing DNA and mRNA molecules. For example, lncRNA APOLO can dynamically regulate the cyclization process of the *PID* promoter to influence the expression of the *PID* gene. They can also trans-regulate the expression of several distal auxin response genes, such as *WAG2* (a gene encoding kinases involved in auxin transport) and *AZG2*, by binding to target DNA to form R loops (RNA:DNA hybrids), thereby influencing the formation of lateral roots [16]. In *A. thaliana*, the cold-induced lncRNA SVALKA (SVK) is reverse- transcribed downstream from the *CBF1* gene to produce the antisense lncRNA as *CBF1*, resulting in RNAPII collision at the *CBF1* site, thus inhibiting the production of its full-length mRNA, and thereby limiting the overactivation of cold-response genes [19]. In addition, lncRNA FLORE, the antisense of the circadian rhythm gene CYCLING DOF FACTOR 5 (*CDF5*) [20], and the antisense LncRNAs of the seed maturation gene DELAY OF GERMINATION 1 (*DOG1*) [21,22], and SUPPRESSOR OF FEMINIZATION (*SUF*) [23]. which inhibit the female differentiation regulator MpFGMYB in male moss tissues. These findings indicated that lncRNAs may be involved in regulation of the plant circadian clock, seed maturation and sex differentiation based on similar principles [24]. Besides affecting transcription- and translation-related processes, lncRNAs are also involved in the splicing process [25]. LncRNAs regulate gene expression patterns through interaction with chromatin modification complexes, DNA methylases, histone modification enzymes, etc., thereby affecting plant physiology and development process.

## 3. Regulatory Role of lncRNAs in Perennial Plant Development

Recent research has increasingly focused on the regulatory functions of lncRNAs in various developmental processes in woody plants, particularly wood formation [26], seasonal cambium activity [27], leaf development, floral bud development, and fruit development (Table 1 and Figure 2).

### 3.1. Wood Formation

Wood formation in trees is a complex developmental process arising from the vascular cambium. Cambial stem cells undergo seasonal growth and division, followed by differentiation of xylem mother cells into specialized cell types, primarily tracheids and fibers. A critical phase is secondary cell wall (SCW) biosynthesis, where massive deposition of cellulose microfibrils, cross-linking of hemicelluloses (like xylan), and the synthesis of phenolic the polymer lignin occur. LncRNAs have emerged as crucial regulators within this framework through epigenetic, transcriptional, and post-transcriptional mechanisms.

Twelve lncRNAs that regulate sixteen genes related to xylogenesis (wood formation) processes, such as cellulose and lignin synthesis, plant hormone control, etc., were identified in *Populus tomentosa* [4]. More lncRNAs were differentially expressed in mature xylem than in developing xylem, suggesting that the regulation of lncRNAs is affected by transcriptional rearrangement during wood biosynthesis. The lncRNA TCONS_00078539 has been identified as a potential target for miR168, which may be involved in auxin signaling and secondary cell wall biosynthesis [28]. In addition, a total of 7655 stably expressed lncRNAs were identified from gibberellin-treated and controlled *Populus sinensis* leaves, among which 410 were correlated with gibberellin expression levels. The prediction of the potential target genes of these lncRNAs by computational analysis revealed that 939 were cis-regulated and 965 were trans-regulated. Among them, there were genes related to auxin signaling pathway, and cellulose and pectin synthesis. Additionally, seven lncRNAs were predicted to be targets for eighteen miRNAs [29].

For other lncRNAs involved in wood formation, the expression levels of the lncRNA NERDL and the gene *PtoNERD* are highly correlated, and their single nucleotide polymorphisms (SNPs) are significantly associated with wood formation traits [30]. *PtoNERD* is a key gene encoding a protein involved in the RDR2 (RNA-dependent RNA polymerase 2)-independent DNA methylation pathway, which is guided by a 21-nt siRNA and mediates the de novo methylation of the newly integrated transposon, thereby silencing gene expression [31]. The promoter regions of the lncRNA NERDL and *PtoNERD* partially overlap and may regulate their expression at the transcriptional or epigenetic level. Tissue expression analysis revealed that the expression level of lncRNA NERDL was the highest in the phloem, suggesting that it has a regulatory role in secondary growth. Moreover, both the lncRNA NERDL and *PtoNERD* are highly expressed in the xylem, further indicating the crucial functions in wood formation. Genetic interaction analysis indicates that lncRNA NERDL and *PtoNERD* have a synergistic regulatory effect on certain traits, such as breast diameter (D) and fiber width (FW), suggesting that their genetic interaction directly affects the wood formation process. These findings reveal that the molecular mechanism by which the lncRNA NERDL participates in the RDR2-independent DNA methylation pathway is by regulating the expression of *PtoNERD*, thereby influencing wood development [30].

Lignin plays a key role in wood formation by enhancing the rigidity and stability of cell walls, providing mechanical support for plants, and promoting the sturdiness and structural integrity of wood. In poplar, 124 significant associations were found among 30,265 single nucleotide polymorphisms (SNPs) derived from 203 lignin synthesis genes, 81 transcription factor (TF) genes, 36 miRNA genes and 71 lncRNA loci, including 10 growth and wood-characteristic traits. These genes play a common role in wood formation [32], which provides new insights into the lignin biosynthesis pathway in poplar and enables the use of novel genetic factors as biomarkers to facilitate the genetic improvement of trees.

Trees in temperate and boreal regions undergo annual cycles of growth and dormancy via the vascular cambium. An analysis of newly identified lncRNAs and circRNAs found that 2037 lncRNAs and 299 circRNAs were differentially expressed during the vascular cambium annual cycles. By aligning miRNA precursors to the 7655 lncRNAs, 21 lncRNAs were identified as precursors of 19 known miRNAs. Notably, circRNA103 and MSTRG.10851.1, which regulate the cambium periodicity, may interact with miR482, shedding new light on the regulation of the activity-dormancy cycle [27]. It was investigated that the dormancy release process of trees may have similarities to the vernalization of *Arabidopsis*, as both processes require prolonged cryogenic treatment [33]. Through comprehensive multi-omics studies, Hu et al. [34] identified a particular lncRNA named Phenology Responsive Intergenic lncRNA 1 (PRIR1), which is specifically expressed at the bud germination stage, and overexpression of PRIR1 in hybrid poplar can induce earlier bud germination. In addition, a genome-wide transcriptome analysis suggested that PRIR1 may promote germination by inhibiting genes associated with dormancy maintenance and promoting genes associated with active growth. During dormancy release, the level of DNA methylation on the PRIR1 promoter is reduced, thus activating PRIR1 expression. PRIR1 may directly activate *PtEXL5* expression by altering the local chromatin state, thereby promoting bud break, which may contribute to cell expansion when plants resume growth after dormancy release [34].

### 3.2. Adventitious Root Development

Adventitious root formation is essential for vegetative propagation of economically important woody plant species. A better understanding of the genetic and physiological mechanisms that influence adventitious root formation will improve the success of the commercial deployment of trees. Although many lncRNAs have been identified in plants based on the rapid development of high-throughput sequencing technology in recent years, adventitious root formation in woody plants remains largely unexplored.

Liu et al. systematically identified and identified 5952 putative lncRNAs expressed at different developmental stages of poplar adventitious roots on a genome-wide scale. It was found that *lncWOX5* negatively regulated *WOX5*, while *lncWOX11* positively regulated *WOX11*. These findings, as well as the characterization of *lncWOX5* and *lncWOX11*, provided a better understanding and enabled the preliminarily establishment of a theoretical framework for adventitious root formation and development [35]. In addition, Ran et al. cloned and characterized the novel lncRNA molecule *lncWOX11* through sequence alignment, and they found that the overexpression of *lncWOX11a* led to a decrease in the number of adventitious roots on the cuttings of transgenic poplars. In addition, *cis*-regulatory module prediction and CRISPR/Cas9 knockout experiments with poplar protoplasts showed that *lncWOX11a* acts as a negative regulator of adventitious root formation by downregulating the WUSCHEL-related homeobox gene *WOX11*, which is believed to activate adventitious root development in plants. Collectively, these findings imply that *lncWOX11a* is essential for regulating the formation and development of adventitious roots [36].

### 3.3. Leaf Development

Leaves are vital plant organs due to their roles in photosynthesis. It has been found that the photosynthetic efficiency is significantly affected by non-coding RNAs (ncRNAs), which play a key regulatory role in the allelic variation in photosynthesis- genes. In poplar, 30 photosynthesis-related genes that may share the same regulatory pathway were identified by co-expression network analysis. Upon combining this analysis computer prediction, expression pattern analysis, and degradation group sequencing, tweve lncRNAs and six miRNAs were found to be associated with the photosynthetic co-expression network of poplar [37].

In *Liriodendron chinense*, lncRNAs, miRNAs, and TFs are were found to be very crucial for the development of leaves, and various lncRNA-TF regulatory modules were found to participate in leaf polarity establishment and leaf morphology regulation, such as lch-lnc6026-BLH2, lch-lnc0809-ATHB4, lch-lnc4261/5500-GRF1, lch-lnc5465-bHLH30, lch-lnc2601/3102/6972-TCPs, and lch-lnc1857/4867/6438-AUX/IAAs [38].

Heterophylly is a phenomenon in which a single plant produces leaves of different shapes and forms depending on environmental conditions. High-throughput RNA sequencing (RNA-seq) analysis revealed that lncRNAs were differentially expressed in heteromorphic leaves of *Populus euphratica*. A total of 36,492 genes and 1725 lncRNAs were detected, among which 586 genes and 54 lncRNAs were differentially expressed [39]. A previous study also showed that several lncRNAs that play important roles in *Ginkgo biloba* leaf development were associated with photosynthesis, plant hormones, and secondary metabolism [40], while another discovered that lncRNAs regulated flavonoid biosynthesis [41].

### 3.4. Flower Bud and Female Bulb Development

It is known that lncRNAs can directly target TFs to regulate leaf and flower development. For example, COLD-ASSISTED INTRONIC NON-CODING RNA (COLDAIR) is a lncRNA that can repress the expression of FLOWER LOCUS C (FLC) to regulate flowering [42]. A novel lncRNA called PRIR1 (long-chain non-coding RNA1 between phenology-related genes) has been found to play a key role in bud opening-activation. Preliminary mechanism studies suggest that PRIR1 may promote bud opening by activating its neighboring gene, *PtEXL5* (EXORDIUM-LIKE 5), which is the activator of bud opening [34]. In female *G. biloba*, 4075 differentially expressed lncRNAs were found between the initial differentiation and early vigorous differentiation of the stalk primordium. Additionally, 160 differentially expressed lncRNAs were found between the early and late stages of differentiation.

In the initial and exuberant differentiation stages of female stalk primordia, 362 lncRNAs mediated transcriptional regulation through cis-regulation, two of which functioned through trans-regulation, while a variety of lncRNAs participated in the formation of 10,248 lncRNA-miRNA-mRNA as ceRNAs [43]. In *Liriodendron chinense*, the miRNA-lncRNA-TF regulatory networks contained 105 miRNAs, 258 lncRNAs, and 393 TFs, and 22 endogenous target mimics were constructed. Notably, lch-lnc7374-miR156h-*SPL3* and lch-lnc7374-miR156j-*SPL9* were found to be potential regulators of stamen and pistil development in *L. chinense*, respectively [38]. In *Catalpa bungei*, 680 differentially expressed genes and 817 differentially expressed lncRNAs were detected during the initiation of floral transition [44]. These studies suggest that it is necessary to investigate the role of lncRNA-mediated seasonal variation in perennial plants.

### 3.5. Fruit Development

The roles of lncRNAs in fruit development and secondary metabolism, especially through the regulation of the biosynthesis of phenylpropanoid metabolites, have also been extensively investigated. For example, Zhang et al. identified 118 differentially expressed lncRNAs during the development of sea buckthorn fruits, and found that these lncRNAs were mainly enriched in the biosynthesis of ascorbic acid, carotenoids and flavonoids, among which two lncRNAs, namely LNC1 and LNC2, were important regulatory factors of anthocyanin biosynthesis [45]. However, these two lncRNAs perform opposite functions; in sea buckthorn fruit, LNC1 upregulates the expression of the miRNA156 target gene *SPL9* by trapping miRNA156, thus promoting anthocyanin biosynthesis in sea buckthorn fruit, while LNC2 upregulates the expression of the miRNA828 target gene *MYB114* by trapping miRNA828. As a result, anthocyanin biosynthesis in sea buckthorn fruit decreased [45]. In *Malus pumila*, the lncRNA *MdLNC499* was found to be involved in early anthocyanin accumulation, and induced the expression of ethylene response factor (ERF) protein ERF109; moreover, the MdERF109 protein directed the binding of anthocyanin-related gene promoters, promoting apple coloring [46]. In *Malus* × *domestica*, studies have found that two lncRNAs act as eTMs of miR156a, which prevent miR156a from cleaving SPL2-like and SPL33 to regulate anthocyanin accumulation [47].

**Table 1:** Regulatory role of lncRNAs in perennial plant development.

Species	Name	Developmental Stage or Tissue	Function	Molecular Mechanism	References
*P. tomentosa*	NERDL	Cambium and Xylem	Secondary growth and wood formation	Modulates wood formation by directly or indirectly interacting with PtoNERD mRNA, thereby regulating its transcription or translation	[30]
*P. deltoids* × *P. euramericana* cv. ‘Nanlin895′	*lncWOX5* and *lncWOX11*	Adventitious roots	The formation and development of adventitious roots	*lncWOX5* negatively regulated WOX5 and *lncWOX11* positively regulated WOX11	[35]
	*lncWOX11a*	Adventitious roots	Suppresses adventitious root formation	*lncWOX11a* acts as a negative regulator of adventitious rooting by downregulating the WUSCHEL-related homeobox gene *WOX11*, which is supposed to activate adventitious root development in plants.	[36]
*L. chinense*	lch-lnc6026, lch-lnc0809, lch-lnc4261/5500, lch-lnc5465, lch-lnc2601/3102/6972, lch-lnc1857/4867/6438	Leaf	Leaf polarity establishment and leaf morphology modulation	miRNA-lncRNA-TF regulatory networks	[38]
	lch-lnc7374	Flower	Stamen and pistil development	miRNA-lncRNA-TF regulatory networks	[38]
*G. biloba*	MSTRG.2203.13-Gb_Novel_miR190-*Gb_35828*	Female stalk primordia	Involved in the initial and exuberant differentiation stages of female stalk primordia	lncRNAs mediated transcriptional regulation through cis-regulation, trans-regulation, lncRNA-miRNA-mRNA as CeRNAs	[43]
*C. bungei*	LXLCO_019079, LXLOC_017817 and LXLOC_030659	Axillary buds	Initiation of floral transition	lncRNA-mRNA interaction pairs may participate in floral transition	[44]
*H. rhamnoides* L. subsp. *Mongolica Rousi* × *Chinensis Rousi*	TCONS_00694050 (LNC1)	Mature green (MG), breaker (BR) and red-ripe stage of fruit	Anthocyanin biosynthesis, regulation of fruit quality	Functions as a molecular decoy that sequesters miR156, thereby releasing suppression of its target gene *SPL9* and enhancing anthocyanin biosynthesis in fruits.	[45]
	TCONS_00438839 (LNC2)	Mature green (MG), breaker (BR) and red-ripe stage of fruit	Anthocyanin biosynthesis, regulation of fruit quality	Acts as a molecular decoy that sequesters miR828, thereby derepressing its target gene *MYB114* and suppressing anthocyanin biosynthesis in fruits.	[45]
*M. pumila*	MdLNC499	Apple fruit peels and callus	Anthocyanin accumulation, promoting apple coloring	Functions as a scaffolding molecule that facilitates the assembly of TFs MdWRKY1 and MdERF109 into a regulatory complex, which activates anthocyanin biosynthetic genes to enhance apple fruit coloration.	[46]
*Malus* × *domestica*	MLNC3.2 and MLNC4.6	Apple fruit peels	Anthocyanin accumulation	Preventing miR156a from cleaving SPL2-like and SPL33 to regulate anthocyanin accumulation	[47]

## 4. Stress Regulation by lncRNAs in Perennial Plants

Plants are often exposed to biotic or abiotic stresses that are harmful to their development and survival, such as pathogen infection, extreme temperature, drought, and high salt. To counter such stresses, plants have developed survival strategies involving lncRNAs, which tend to be stress responsive as well as spatially and temporally specific in terms of their expression. Therefore, lncRNAs are thought to act as effectors during stress responses [48]. Recent studies have shown that the genetic transformation of ncRNAs and their target genes can change the plants’ phenotypes and tolerance to abiotic [49,50,51] and biotic [52] stresses in plants. In general, lncRNAs affect the abiotic stress response by recruiting complex mechanisms based on eTMs, antisense transcription-mediated regulation or chromatin regulation, or by directly regulating the transcription of various abiotic stress-responsive genes [53]. Forest trees are affected by climate change, anthropogenic pressure, and abiotic and biotic stresses [54]. To date, conventional tree breeding has been limited to enhancing overall productivity, and our understanding of the genetic basis of their quantitative traits is still inadequate [55].

### 4.1. Regulation of the Response to Biotic Stress by lncRNAs

When pathogens and pests infect plants, protective immune responses are elicited, in which lncRNAs play important roles, and regulate gene expression through various mechanisms to enhance the plants’ biological stress resistance. In crops, *Liberibacter asiaticus* can induce immune responses by infecting the phloem of citrus, resulting in citrus greening disease [56]. A total of 8742 lncRNAs were identified by high-throughput sequencing, including 2529 novel lncRNAs, some of which were found by SNP analysis to be significantly related to citrus greening disease. The lncRNA LNC_28805 was co-expressed with several defense-related genes, and was predicted to be targeted by miRNA5021. LNC_28805 is a lncRNA that may compete with endogenous miR5021 to maintain the expression homeostasis of pathogenicity response genes [57]. In *Actinidia chinensis* infected with *Pseudomonas syringae*, an identified lncRNA formed a complex secondary structure, which was co-expressed with protein-coding genes related to multiple plant defense processes, including the innate immune response, systemic acquired resistance, and salicylic acid-mediated defense [58]. In addition, expression of the lncRNA LRR-AS was induced in leaves infected with phytoplasma in *Morus alba*. This lncRNA exhibits high complementarity to the serine/threonine-protein kinase gene sequence of an LRR receptor in mulberry and may function as a natural antisense transcript (NAT) to post-transcriptionally suppress its expression.

In summary, plant responses to pathogen attacks depend on pathogen recognition at the cellular level, and lncRNAs, as targets of small RNAs, inhibit the function of corresponding small RNAs, triggering a complex defense signaling network at the molecular level to coordinate transcriptional reprogramming.

### 4.2. Regulation of the Response of lncRNAs to Abiotic Stress

**Figure 3:**
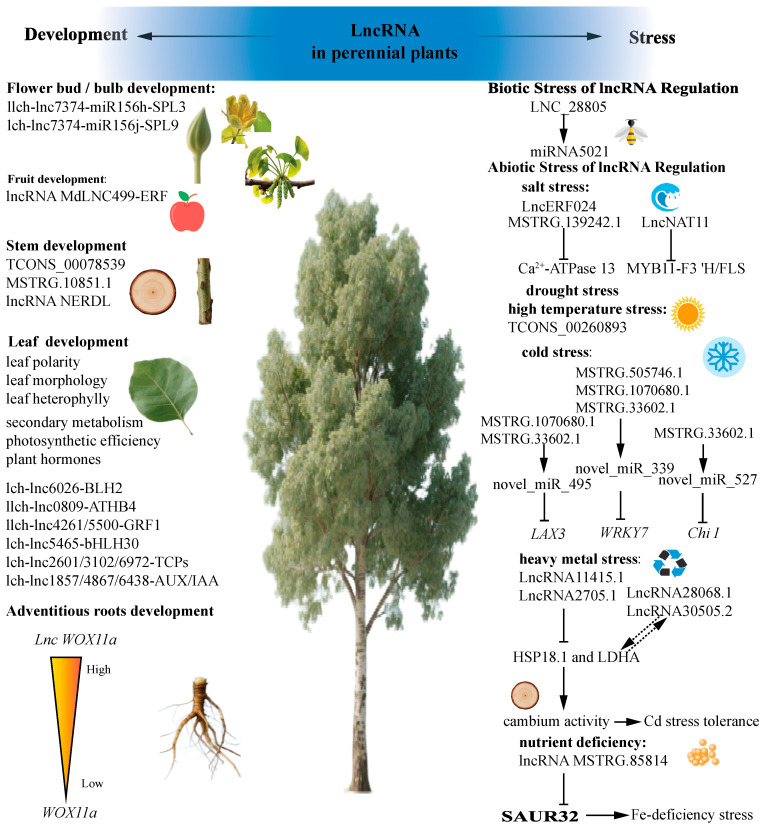
Long non-coding RNAs are involved in perennial plant growth and development as well as adverse stress.

Plants are exposed to various stresses in their environment, and increasing evidence suggests that lncRNAs are involved in the stress responses of multiple species [59,60,61,62] (Table 2 and Figure 3). Here, by compiling an array of studies, we highlight not only the individual roles of these lncRNAs but also their synergistic interactions with one another.

#### 4.2.1. Response to High-Salt Stress

Soil salinization, or excessive accumulation of salts in the soil, is one of the major environmental and socioeconomic problems worldwide that is threatening the sustainable development of agricultural ecology around the world. Saline-alkali land not only reduces soil fertility, but also affects the normal growth of plants, resulting in a decline in agricultural productivity. lncRNAs play an important regulatory role in the mechanisms by which woody plants cope with high salt stress. Previous studies have identified several lncRNAs associated with high salt stress, which improve plant salt tolerance by regulating gene expression, enzyme activity and ion transport [63,64,65,66,67,68].

In addition, tissue-specific and species-specific responses to salt stress have been reported in two poplar species. The study found that 322 lncRNAs were highly expressed in *P. euphratica* but not in *P. alba* var. pyramidalis, and the species-specific responses of 3425 lncRNAs to high salt stress were identified in *P. euphratica* [63].

In tea plants, there were 172 lncRNAs were found to be differentially expressed under high NaCl stress. Among them, lncRNA MSTRG.139242.1 may interact with TEA027212.1 (Ca^2+^-ATPase 13), indicating that it participates in Ca^2+^ transport and alleviates the harmful effect of salt on cells [64]. In *P. tomentosa,* overexpression of the antisense transcript lncERF024 was found to enhance the salt tolerance, the mechanism of which was related to the photosynthesis and stress response pathways, which may be involved in the regulating the expression relevant genes and function of proteins [65]. In addition, 1183 lncRNAs were found to be differentially expressed in *P. trichocarpa* under salt stress, among which *Ptlinc-NAC72* sensitized the plant to salt stress and negatively affected salt tolerance [66].

It has also been found that some lncRNAs in *Pistacia vera* play important roles in adaptation to high salt concentrations, and can regulate *MYB*, *NAC*, *WRKY*, *bZIP*, *TCP*, *GRAS* and other TFs and transporters at the transcriptional and post-transcriptional levels, as well as *ATPase*, *LEA*, *Laccase*, *CERK1*, *UGT* and other functional genes involved in hormone signaling pathways [67].

Flavonols are important secondary metabolites in plant resistance to environmental stress. In *G. biloba*, *GbMYB11* plays a key positive regulatory role in flavonol biosynthesis. LncNAT11, the antisense lncRNA complement to *GbMYB11*, negatively regulates flavonol biosynthesis by inhibiting *GbMYB11* expression. In addition, the LncNAT11-MYB11-F3′ H/FLS module enhances the salt tolerance of *G. biloba* by regulating flavonol biosynthesis and increasing the efficiency of reactive oxygen species removal [68].

#### 4.2.2. Response to Drought Stress

Drought is one of the major abiotic stresses encountered by plants and can have many negative effects on them. In terms of morphological structure, drought stress can lead to smaller and thicker leaves, stomatal closure, and root development, to reduce water evaporation and improve water absorption. In terms of photosynthesis, drought stress can reduce photosynthetic efficiency and chlorophyll content, thus affecting the synthesis of photosynthetic products. For membrane systems, drought stress can increase membrane permeability and affect membrane integrity, and previous studies have shown that lncRNAs play an important role in the response of woody plants to drought stress [69,70,71].

In *Betula platyphylla*, 53 lncRNAs associated with drought stress were identified, and 6 were selected for further study. Overexpression of four lncRNAs enhanced drought resistance, and the remaining two increased drought sensitivity. In *Hevea brasiliensis*, 1244 lncRNAs were found to be associated with drought stress, which were related to the synthesis of secondary metabolites, the glucose metabolism pathway, plant hormone signal transduction and other pathways [69]. In *P. trichocarpa*, 504 lncRNAs were found to be specifically expressed under drought stress, and they play a regulatory role as miRNA targets. In addition, similar differential expression was also found under low-temperature and waterlogging stresses [70].

#### 4.2.3. Response to High Temperature Stress

Global warming is affecting tree growth and forest productivity. Thus, to protect forest ecosystems and ensure the sustainable development of the wood industry, it is important to conduct in-depth research on the response mechanisms by which of plants respond to high temperature, mine the gene loci of species with high-temperature tolerance, and cultivate the woody plant varieties of high-temperature tolerance through gene editing and other techniques are of great significance for coping with the challenges brought by global warming, to protect the forest ecosystem and ensure the sustainable development of the wood industry.

In addition, 204 high-temperature-responsive lncRNAs have been identified in *Populus simonii*, among which TCONS_00202587 binds to upstream sequences through its secondary structure and interferes with target gene transcription, and TCONS_00260893 promotes the influx of Ca^2+^ influx into cytoplasm under heat stress by regulating the proportion of target gene transcriptional variants [72]. These findings demonstrate that lncRNAs regulate target genes through RNA scaffolding mechanisms or RNA interference pathways.

In *Populus qiongdaoensis*, 1690 genes, 25 lncRNAs and 15 miRNAs were found to be differentially expressed in seedlings exposed to temperatures of 40 °C, and these target genes were associated with cell membrane stability, plant hormone signal transduction, and antioxidant and uronic acid metabolism. By studying the miRNA-lncRNA-mRNA network, it was found that miRNAs may negatively regulate lncRNAs and mRNAs in response to heat stress, and lncHSP18.2 in particular may cis- regulate HSP18.2 in response to high temperature stress [73]. A lncRNA gene interaction network constructed for *Populus* × *canadensis*, revealed that lncRNAs can respond to heat stress by regulating heat shock protein (HSP) family genes [74]. The potential lncRNAs and HSP family genes identified represent a genetic resourcefor improving our understanding of the heat-adaptation mechanisms of trees.

In *Ziziphus jujuba*, it was found that 8260 lncRNAs may be possibly related to high-temperature stress. A bioinformatics analysis of potential target mRNAs of cis-acting lncRNAs showed that multiple differentially expressed mRNAs co-located with differentially expressed lncRNAs were highly enriched in pathways related to stress response and regulation of metabolic processes, suggesting that lncRNAs may interact with target mRNA in response to high-temperature stress [75].

#### 4.2.4. Response to Cold Stress

Low-temperature stress is the important environmental factor affecting plant growth and development as well as crop yields, with the global decline in crop yields caused by low temperatures becoming increasingly serious. Therefore, studying how plants perceive and respond to low-temperature stress and how to improve their cold tolerance of plants has become an important research topic in plant physiology and molecular biology. COLDAIR is the first identified plant lncRNA involved in the epigenetic modification of gene chromatin. It is a transcript from the first intron of the *A. thaliana* FLC gene, which is about 1100 nt long and has a 5′ cap but is not polyadenylated at the 3′ end.

In *Manihot esculenta*, high-throughput RNA-seq analysis identified 316 lncRNAs associated with low-temperature stress, among which CRIRIR was found to interact with the cold shock protein calmodulin, which improved its translation efficiency under low-temperature conditions and enhanced the cold tolerance of plants [76]. In *Picea glauca*, at low temperatures the expression level of the novel miRNA 495 decreased, while that of the *LAX3* gene increased. It was also found that the lncRNAs MSTRG.505746.1, MSTRG.1070680.1, and MSTRG.33602.1 might bind to pre-novel_miR_339 to promote the expression of *WRKY7* genes in the stress response. Additionally, *LAX3* could be protected by the lncRNAs MSTRG.1070680.1 and MSTRG.33602.1 by acting as sponges for novel_miR_495 to initiate auxin signal transduction. Moreover, the lncRNAs MSTRG.505746.1, MSTRG.1070680.1, and MSTRG.33602.1 might act as sponges for novel_miR_527 to enhance the expression of *Chi I* in early somatic embryo development [77].

The expression dynamics of lncRNAs in *Vitis vinifera* subjected to cold stress were investigated by high-throughput RNA-seq analysis. The analysis showed that, in grapevines subjected to cold treatment, there was a remarkable increase in the expression of 203 identified lncRNAs, while the expression of 144 known lncRNAs was significantly decreased. In addition, the same study identified 2088 previously uncharacterized lncRNA transcripts in cold-exposed *V. vinifera*, of which 284 and 182 novel lncRNAs were found to be markedly upregulated and downregulated, respectively. Additionally, 242 differentially expressed lncRNAs in *V. vinifera* were predicted to have a cis-regulatory relationship with 326 protein-coding genes [78]. These studies will provide new insights for sustainable plant production and addressing environmental stress.

#### 4.2.5. Response to Heavy Metal Stress

Plants respond to heavy-metal stress through various mechanisms, and lncRNAs play important roles in this response. In *P. tomentosa*, 56 lncRNAs expressed specifically under lead stress and 226 differentially expressed lncRNAs were identified, and their target genes and signaling pathways were predicted. In particular, an analysis of the lncRNA PMAT and its interacting target gene *PtoMYB46* (homologous to *Arabidopsis AtMYB46*) revealed that *PtoMYB46* could promote the growth of poplar and improve its absorption capacity. PMAT belongs to the class of NAT lncRNAs, and it is transcribed from the antisense chain of the *ProMATE* (Pb^2+^-induced multidrug and toxic compound extrusion (MATE)) gene, enabled by the stability of the formed PTOMYB46-PMAT-MATE pathway [79].

Cd-responsive lncRNAs and lncRNA-mediated genetic networks for photosynthesis and leaf physiology were revealed in *P. tomentosa*. In particular, in the two lncRNA-gene pairs, MSTRG.22608.1-*PtoMYB73* and MSTRG.5634.1-*PtoMYB27*, *were* detected insertions/deletions were detected within the lncRNAs, which acted as polymorphisms driving target gene expression in Cd tolerance [80], Similarly, lncRNAs and heterologous overexpression of *PtoMYB73* and *PtoMYB27* could enhance Cd tolerancein *Arabidopsis*. These results reveal the mechanism by which this lncRNA may conserved in between annuals and perennials.

In *B. platyphylla*, 30 lncRNAs differentially expressed under cadmium stress were identified, including 16 upregulated lncRNAs and 14 downregulated lncRNAs. The lncRNAs lncRNA28068.1 and lncRNA2705.1 were found to interact with the HSP18.1 and LDHA was found to increase cadmium tolerance, whereas lncRNA11415.1 and lncRNA30505.2, with target genes that encode heat shock protein and L-lactate dehydrogenase, respectively, increased cadmium sensitivity [81].

Additionally, the authors of [82] noted a trade-off between maintaining mineral nutrients and cadmium minimization in safe crop breeding, providing new insights for sustainable plant production and addressing environmental issues.

#### 4.2.6. Response to Nutrient Deficiency

Nutrient deficiency is one of the major environmental factors that limit plant growth and development. Common nutrients in which plants are deficient include deficiencies in elements such as nitrogen, phosphorus, iron, and sulfur, which are essential for plant growth and development. Under nutrient deficiency conditions, plants utilize a variety of physiological and molecular response mechanisms, among which lncRNAs play key roles in adapting to adverse environmental conditions.

It was reported that non-coding RNA modulates the expression of target genes that regulate nitrogen dynamics, and also affect plant growth, yield loss, and the response to pollution stress [83]. In *P. tomentosa*, a total of 126 lncRNAs specifically expressed under low nitrogen stress were identified in the whole genome. Among them, 14 lncRNAs serve as miRNA precursors, 4 interact with miRNA, and 15 are NATs, establishing a regulatory network involved in the response to nitrogen deficiency stress in *P*. *chinensis*. The study also found that the same gene locus can produce different lncRNAs [84], providing more information on low-nutrient adaptation mechanisms in woody plants.

In *M. pumila*, the expression of lncRNA MSTRG.85814 was induced by iron deficiency, and there were 13 splicing variants, one of which positively regulated the mRNA expression of the auxin-upregulating protein gene *SAUR32*, thereby promoting its expression and activating proton-pump acidification of the rhizosphere to promote iron absorption [85]. These results reveal the mechanism by which this lncRNA promotes adaptation to environmental Fe-deficiency stress adaptation.

While each type of stress elicits unique physiological responses, plants have evolved conserved regulatory pathways to integrate stress signals and mount adaptive defenses. In *Pyrus pyrifolia*, the lncRNA *lncABF2* is upregulated under cold and salt stress and interacts with *PpABF2* and other genes to enable cross-tolerance to multiple stresses [60]. LncRNAs often interact with AREBs/ABFs to amplify ABA-mediated stress responses, and this interaction is conserved across stress types. For instance, in *P. tomentosa*, the lncRNA *PtlncABA3* regulates ABA signaling and antioxidant defense while also modulating cambial activity balancing stress tolerance with wood formation [30]. In *Pinus sylvestris*, the lncRNA *PllncMEM1* is induced by cold stress and stabilizes the ABA receptor PlPYL9, creating epigenetic memory that enables the tree to exhibit a faster ABA-mediated cold response. Moreover, the lncRNA PllncAPX1 is upregulated under cold and heat stress [77]. In *Betula platyphylla*, the lncRNA *BplncCAT1* similarly upregulates *BpCAT1* under drought and Cd^2+^ stress, reducing H_2_O_2_ accumulation [81]. Across diverse species, the conserved regulation of core biological pathways, such as ABA signaling and antioxidant defense, is conserved, which achieved through the evolution of lncRNAs with distinct sequences but analogous functions or structural features.

**Table 2:** Stress regulation by lncRNAs in perennial plant.

Species	LncRNA	Stress	Molecular Mechanism	Up/Down-Regulated	Promotes Tolerance/ Confers Sensitivity	References
*Citrus jambhiri*	LNC_28805	Diseases	*LNC28805* might compete with endogenous miR5021 to maintain the homeostasis of immune gene expression levels.	Upregulated	Promotes Tolerance	[57]
*Camellia sinensis*	MSTRG.139242.1	Salt stress	May interacted with TEA027212.1 (Ca^2+^-ATPase 13), participating in Ca^2+^ transport and alleviating the harmful effect of salt on cells	Upregulated	Promotes Tolerance	[64]
*P. tomentosa*	lncERF024	Salt stress	May be involved in the regulation of both gene expression and protein function conferring salt tolerance	Upregulated	Promotes Tolerance	[65]
*P. trichocarpa*	*Ptlinc-NAC72*	Salt stress	Mitigate growth costs by conferring plant resilience to salt stress	Upregulated	Promotes Tolerance	[66]
*G. biloba*	LncNAT11	Salt stress	LncNAT11-MYB11-F3′H/FLS module enhances salt tolerance by regulating flavonol biosynthesis and increasing the efficiency of reactive oxygen species removal	Downregulated	Promotes Tolerance	[68]
*P. simonii*	TCONS_00202587	Heat stress	Binds to upstream sequences via its secondary structure and interferes with target gene transcription	Upregulated	Promotes Tolerance	[72]
	TCONS_00260893	Heat stress	Enhances calcium influx in response to high-temperature treatment by interfering with a specific variant/isoform of the target gene	Upregulated	Promotes Tolerance	
*P. qiongdaoensis*	lncHSP18.2	Heat stress	miRNAs may negatively regulate both lncRNAs and mRNAs in tree responses to heat stress, lncHSP18.2 may cis-regulate HSP18.2.	Upregulated	Promotes Tolerance	[73]
*P.* × *canadensis*	lncRNAPc3, lncRNAPc5, lncRNAPc12, and lncRNAPc14	Heat stress	May cis-regulate HSP genes via co-expression in response to heat stress	Upregulated	Promotes Tolerance	[74]
*Picea glauca*	lncRNAs MSTRG.505746.1, MSTRG.1070680.1, MSTRG.33602.1; MSTRG.1070680.1 MSTRG.33602.1; MSTRG.505746.1, MSTRG.1070680.1, MSTRG.33602.1	Cold stress	Cold stress-enhanced early somatic embryogenesis through long non-coding regulatory network	Upregulated	Promotes Tolerance	[77]
*P. tomentosa*	lncRNA PMAT	Pb^2+^ toxic heavy-metal stress	PMAT-PtoMYB46-PtoMATE-PtoARF2 regulatory module control Pb^2+^ tolerance, uptake, and plant growth	Upregulated	Promotes Tolerance	[79]
	*MSTRG.22608.1* *MSTRG.5634.1*	Cd stress	MSTRG.22608.1 with its cis-target gene PtoMYB73, and MSTRG.5634.1 with its trans-target gene PtoMYB27	Upregulated	Promotes Tolerance	[80]
*B. platyphylla*	*LncRNA28068.1* and *LncRNA2705.1*	Cd stress	*LncRNA28068.1* and *LncRNA2705.1* could confer Cd tolerance	Upregulated	Promotes Tolerance	[81]
	*LncRNA11415.1* *and LncRNA30505.2*		*LncRNA11415.1* and *LncRNA30505.2* conferred sensitivity to Cd.	Downregulated	Confers Sensitivity	
*M. domestica*	lncRNA MSTRG.85814	Nutrient stress	MSTRG.85814.11 was shown to positively promote SAUR32 expression, which then activated proton extrusion involved in the Fe-deficiency response.	Upregulated	Promotes Tolerance	[85]

## 5. Conclusions and Perspectives

The functional delineation of lncRNAs in perennial plants considerably lags behind that in herbaceous model systems, primarily owing to perennial plants’ multifaceted biological intricacies and associated the methodological challenges. A primary limitation lies in the vague functional annotation of most identified lncRNAs. Wood quality engineering leverages lncRNAs to optimize industrially relevant traits. Although transcriptomic analyses in woody plants have revealed thousands of expressed lncRNAs, fewer than 5% have been experimentally validated in terms of their functions [86,87]. Major areas include lncRNA regulation of wood formation and stress tolerance. This knowledge gap stems from a poor mechanistic understanding of how lncRNAs regulate wood-specific processes such as secondary-xylem formation or perennial flowering. In addition, limited sequence conservation among species presents a major barrier to cross-species extrapolation, which is further compounded by technical obstacles. For example, the genetic transformation efficiency of forest trees is low, and RNA extraction from lignified tissues is difficult, resulting in extremely low lncRNA abundance. Concurrently, the absence of dedicated databases for woody plant lncRNAs, despite the availability of comprehensive resources, limits systematic exploration of lineage-specific regulators.

While existing tools like CRISPR and RNA-seq are valuable, the systematic identification of lncRNAs in woody species requires more advanced, tailored omics approaches. Strand-specific RNA-seq across developmental stages (e.g., cambial dormancy-active growth transitions), coupled with long-read sequencing (PacBio/Nanopore), enables full-length isoform reconstruction, and the pre-filtering process must exclude known ncRNAs and transcripts with coding potential using tools like CPC/CNCI, followed by expression thresholding (TPM > 0.5). Furthermore, functional validation hinges on robust genetic tools. Gain-of-function studies use tissue-specific promoters (e.g., wood-specific *DX15*) to overexpress lncRNAs, while loss-of-function approaches use CRISPR/Cas9-mediated knockout or VIGS-based transient silencing. For interaction mapping, RNA-pull down assays can be performed to validate binding sites, and multi-omics integration networks can be constructed by correlating lncRNA-miRNA pairs with phenotypic outcomes [88]. In addition, spatial resolution by fluorescence in situ hybridization (FISH) localizes lncRNAs to subcellular compartments, while single-cell transcriptomics unravel the function of lncRNAs with high cell-type specificity in complex tissues, such as lncRNA activity in specialized niches of cambium cells or resin ducts.

The conservation of lncRNA mechanisms is complex, showing significant variation across species, ranging from model annuals to perennials. LncRNAs can act as endogenous RNAs (ceRNAs) by sponging miRNAs, thereby regulating the expression of miRNA-target gene pairs. For instance, it has been shown that an endogenous lncRNA (IPS1) contains miRNA response elements (MREs) that match miR399, induced due to phosphate starvation in *Arabidopsis* [89]. In tomatoes, lncRNA44664 indirectly regulates the resistance to root-knot nematodes by “adsorbing” miR396 [88], and lncRNA23468 serves as a ceRNA to attract miR482b that modifies NBS-LRR genes infection by *Phytophthora infestans* [90]. Furthermore, LncRNAs’ function as ceRNAs in response to alkaline-salt stress was investigated in rice, and an alkaline-salt stress ceRNA network was constructed with many lncRNA-miRNA-mRNA triplets [91]. In *Ginkgo biloba*, it was revealed that lincRNAs contain TE-derived sequences [68]. All in all, some miRNAs that are conserved between annuals and perennials could be regulated by lncRNAs through a similar ceRNA-based mechanism in response to stress. However, lncRNAs function as competing endogenous RNAs that sequester miRNAs and modulate gene expression. It should be noted that the effective operation of this ceRNA mechanism in vivo is subject to strict stoichiometric requirements, and the physiological contexts in which these requirements are met remain an active area of investigation.

Non-coding RNA regulatory networks help us to understand wood formation and the stress response of trees [92,93,94,95]. LncRNA have the potential to be employed as targets for “climate-proofing” forests by engineering stress-tolerant trees. The positive regulation of lncRNA positively regulates plant-freezing tolerance and pathogen-responsive by lncRNA has also been investigated in model plants and some perennial crops [96,97,98]. In *Arabidopsis thaliana*, expression of ELENA1 is induced after bacterial infection, which promotes the transcription of the defense gene PR1 by binding to the MED19a protein, thereby enhancing disease resistance [99]. It was reported that the lncRNA MtCIR2 positively regulates plant-freezing tolerance with regulating MtCBF/DREB1s expression and glycometabolism in *M. truncatula* [95]. In cotton, lncRNA973 was significantly induced under salt stress, enhancing the plant’s salt tolerance by regulating the sodium/potassium ion homeostasis and antioxidant system. Based on the research, the various regulatory mechanisms work together in a single stress response pathway through an integrated network of crosstalk and hierarchical regulation, where lncRNAs often act as central guides and decoys to orchestrate the response. These lncRNAs modulate stress signaling pathways, control hormonal responses and interact through elaborate crosstalk mechanisms, which could ultimately guide the design of robust climate-resilient perennial plants.

To elucidate the molecular mechanisms of lncRNAs in woody plants’ growth and development, with lncRNAs are expected to play an important role in forestry breeding, germplasm innovation and quality improvement. Additionally, it is crucial to study the response mechanisms by which plants respond to different types of stress to improve their stress resistance and agricultural productivity. Many lncRNAs are versatile and can respond to various kinds of stress. The lncRNA *Mu*Lnc1, isolated from *M. alba*, can respond to both salt stress and drought stress, and can show resistance to gray mold and *Pseudomonas syringae*. The production of siRNA by *Mu*Lnc1 after its cleavage represents *Mu*Lnc1’s primary function, with siRNA being able to silence the expression of calmodulin-like genes in mulnC1 [100]. In future studies, it would also be interesting to explore lncRNA-mediated seasonal variation in perennial plants was also appealing. Therefore, developing a comprehensive understanding of lncRNAs’ wide range of regulatory functions for traits influencing biomass productivity and adaptation would aid in the application of biotechnology to genetically improve forest trees (Figure 1 and Figure 3).
